# Transcriptome and Metabolome Reveal Accumulation of Key Metabolites with Medicinal Properties of *Phylloporia pulla*

**DOI:** 10.3390/ijms252011070

**Published:** 2024-10-15

**Authors:** Ji-Hang Jiang, Qian-Zhu Li, Xing Luo, Jia Yu, Li-Wei Zhou

**Affiliations:** 1 State Key Laboratory of Mycology, Institute of Microbiology, Chinese Academy of Sciences, Beijing 100101, China; jiangjh@im.ac.cn (J.-H.J.);; 2 University of Chinese Academy of Sciences, Beijing 100049, China

**Keywords:** macrofungi, steroids, triterpenoids, transcriptome, metabolome

## Abstract

*Phylloporia pulla*, a macrofungal species in the *Hymenochaetales*, *Basidiomycota*, is known to enhance the nutritional and bioactive properties of rice through co-fermentation; however, its own secondary metabolites are not well understood. In this study, an integrative analysis of transcriptome and metabolome data revealed that the accumulation of steroids, steroid derivatives, and triterpenoids in *P. pulla* peaks during the mid-growth stage, while the genes associated with these metabolites show higher expression levels from the early to mid-growth stages. Weighted gene co-expression network analysis identified several modules containing candidate genes involved in the synthesis of steroids, steroid derivatives, and triterpenoids. Specifically, six key hub genes were identified, along with their connectivity to other related genes, as potential catalysts in converting the precursor lanosterol to celastrol. This study enhances our understanding of the secondary metabolites of *P. pulla* and is essential for the selective utilization of these bioactive compounds.

## 1. Introduction

Macrofungi comprise many edible and medicinal species, such as *Cordyceps* spp., *Ganoderma* spp., *Sanghuangporus* spp. and so on [1]. Their various bioactive properties, such as antioxidant, antitumor and immunomodulation, normally come from the highly diverse metabolites, including polysaccharides, terpenoids and polyphenols [2]. Therefore, macrofungi have the potential to be applied as food supplements to improve nutritional and functional compositions. For example, a macrofungal species *Sanghuangporus sanghuang* has been separately fermented with seven grains [3], while ten species from the macrofungal genera *Ganoderma*, *Phylloporia* and *Sanghuangporus* were screened for rice fermentation [4]. Although all these fungal species can improve the quality of grains to some extent, the bioactive metabolites from fungi themselves, especially in the genus *Phylloporia*, remain underexplored [5].

*Phylloporia*, belonging to *Hymenochaetales*, *Agaricomycetes*, is a worldwide wood-inhabiting fungal genus. Most species of *Phylloporia* are weak plant pathogens with host specificity [6]. Over 30 species of *Phylloporia* have been recorded in China [7]. The fermented mycelia of *Phylloporia* were permitted as a new resource food by the National Health Commission of the People’s Republic of China in 2013. Additionally, several patents related to *Phylloporia* as dietary supplements in functional foods are currently in process, with at least one patent authorized by China National Intellectual Property Administration (No. ZL201010579919.2). While the safety of *Phylloporia* has been proven in animal experiments [8], its primary metabolites, viz. saccharides of *Phylloporia,* show a series of bioactive properties, including antioxidant, antitumor, immunomodulation and neurotrophy [9,10,11,12]. Compared with other macrofungi, such as *Ganoderma* [13] and *Sanghuangporus* [14], whose metabolites have been researched and extensively studied using omics approaches, the current knowledge of the secondary metabolites of *Phylloporia* remains limited and requires further comprehensive investigation. 

It is well recognized that traditional methods for isolating and evaluating the activity of secondary metabolites are not efficient for high-throughput analysis of macrofungi’s bioactive properties [15,16]. By contrast, a metabolomic method has the power to reveal a series of secondary metabolites simultaneously. Moreover, an integrative analysis of transcriptome and metabolome data can elucidate the regulatory genes and biosynthesis pathways of key bioactive secondary metabolites [17,18], which could be further applied in the field of synthetic biology. Therefore, integrative analyses of transcriptome and metabolome are essential for unlocking the medicinal values of *Phylloporia*.

Notably, almost all previous medicinal studies and patents related to the medicinal properties of *Phylloporia* refer to the species as *Phylloporia ribis*. However, this species is not distributed in China [19], suggesting that the materials used in China should be another morphologically related species of *Phylloporia*. Our previous research has shown that the fungal fruiting bodies used locally in Shandong Province, China, comprise a mixture of *Phylloporia fontanesiae*, *P. lonicerae*, and *P. pulla*, as determined through fieldwork, morphological examinations, and molecular phylogenetic analyses [19]. Since the fruiting bodies of *Phylloporia* grow slowly under natural conditions, fermented mycelia offer an alternative source for utilizing *Phylloporia*. Among the three identified medicinal species, *P. fontanesiae* and *P. lonicerae* exhibit much stronger host specificity than *P. pulla* [6], making their mycelia challenging to cultivate on a large scale under artificial conditions. This ecological characteristic makes *P. pulla* the most suitable species of *Phylloporia* for medicinal development and utilization.

In this study, the first whole genome sequencing of the genus *Phylloporia* was performed for *P. pulla*. Using this genomic data as a reference, we performed an integrative analysis of the newly generated transcriptome and metabolome of *P. pulla* mycelia at various growth stages under optimal liquid fermentation conditions. The omics data, combined with experimental validation, significantly enhances our understanding of the medicinal properties of *P. pulla*, aiding in the selective development of a functional food.

## 2. Results

### 2.1. Genome Features

A total of 5.09 Gb clean data and 527,905 reads with an average length of 10,361 bp were generated using the PacBio Sequel platform. The genome of *P. pulla* was assembled into 38,086,920 bp, consisting of 68 contigs with the N50 length of 1,385,281 bp and GC content of 49% after correction using the Illumina short reads. Finally, a total of 10,207 protein-coding genes were predicted with an average gene length of 1542 bp.

### 2.2. Gene Expression Profiles at Different Growth Times

To elucidate the gene expression patterns during liquid fermentation process of *P. pulla*, RNA-seq was performed on 12 samples collected at four growth time points. After filtering out the adaptor and low-quality sequences, each library yielded between 37,320,162 and 60,237,772 clean reads, which were mapped to the *P. pulla* reference genome with alignment ratios ranging from 95.19% to 97.85%. 

**Figure 1:**
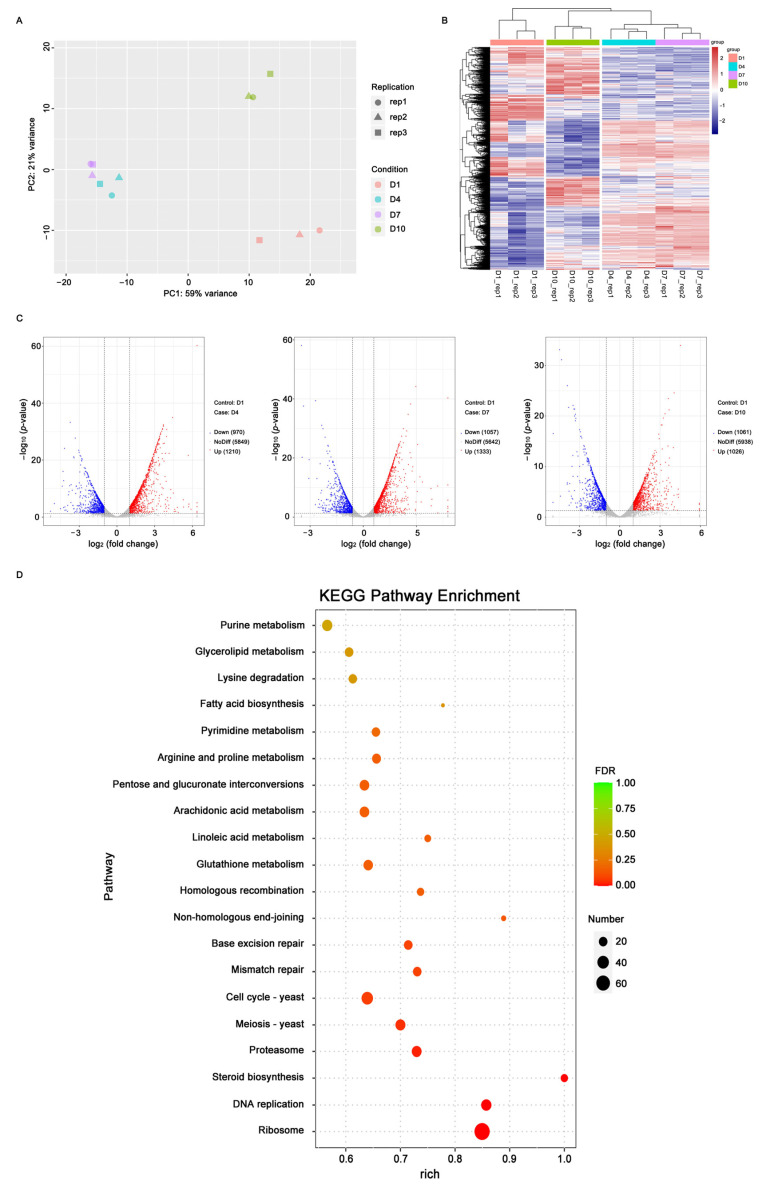
Differentially expressed genes (DEGs) of 12 samples at four growth times of liquid fermentation. (**A**): Principal component analysis of DEGs identified from the 12 samples at four growth times. (**B**): Heatmap of 4351 DEGs. The abscissa represents the hierarchical clustering of 12 samples at four growth times, and the ordinate represents the hierarchical clustering of DEGs. Red color indicates a high expression level, and purple color indicates a low expression level. (**C**): Volcano plots showing the down-regulated (Down), up-regulated (Up) and not changed (NoDiff) genes, respectively, indicated with blue, red and grey colors between D1 and another three growth times (D4, D7, and D10). (**D**): Top 20 enriched KEGG pathways from 4351 differentially expressed genes. The closer the value of false discovery rate (FDR) is to zero, the more significant the enrichment of KEGG pathway is. The circular radius indicates the gene number.

A PCA based on log_2_ values of FPKM demonstrated that biological replicates clustered according to their sampling times, with transcriptomes at D4 and D7 showing the closest relationship (Figure 1A). The DEGs exhibited varied expression patterns, with the DEGs at D4 and D7 showing a closer relationship (Figure 1B). The DEGs between D1 and the other three growth times were identified using a log2 fold change cutoff of >1 (up-regulated) or <−1 (down-regulated), revealing 2180, 2390, and 2087 DEGs between D1 and D4, D1 and D7 and D1 and D10, respectively (Figure 1C). These findings support the appropriateness of the selected sampling times and confirm the high reliability of the transcriptomic data obtained from the 12 samples. 

All 4351 identified DEGs were analyzed against the KEGG databases using BLASTX (*e*-value ≤ 1 × 10^−5^). The top two enriched KEGG pathways were ribosome biogenesis (ko03010, *p*-value = 4.95 × 10^−15^) and DNA replication (ko03030, *p*-value = 1.34 × 10^−16^), both of which are critical for maintaining basal metabolic processes. Additionally, steroid biosynthesis (ko00100) was significantly enriched with a *p*-value of 1.97 × 10^−5^ (Figure 1D); 11 DEGs associated with this pathway were identified (Table S2). Among these, A09727 and A09829, encoding SQLE and FDFT1 respectively, were also differentially expressed in the sesquiterpenoid and triterpenoid biosynthesis pathways (ko00909).

### 2.3. DEGs Involving the Steroid and Terpenoid Biosynthesis

**Figure 2:**
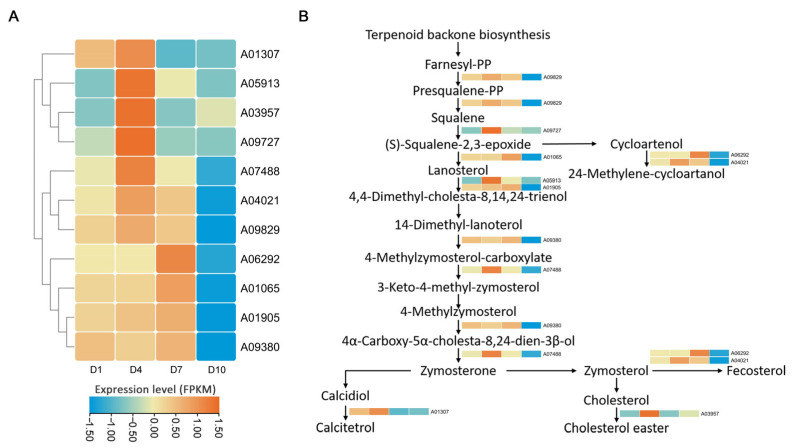
(**A**): Expression patterns of DEGs involving the steroids biosynthesis pathway. The abscissa represents the four growth times, and the ordinate represents the hierarchical clustering of DEGs. Orange color represents a high expression level, and blue color corresponds to a low expression level. (**B**): The steroids biosynthesis pathway with related DEGs and their expression levels at D1, D4, D7 and D10.

The expression pattern of identified DEGs involved in steroid biosynthesis were clustered, generally showing a higher expression level at D4 and D7 (Figure 2A). The proposed steroid biosynthesis pathway of *P. pulla* was constructed by mapping the DEGs to the given pathway (map00100) in the KEGG database (Figure 2B). Similar to the putative pathway in other eukaryotes, the gene A09829, encoding FDFT1 [EC:2.5.1.21], catalyzes the conversion of bimolecular farnesyl-PP (farnesyl pyrophosphate) to squalene, a precursor of triterpenoids. A09829 exhibited higher expression levels at an early growth stage, particularly at D4. Under the catalysis of SQLE [EC:1.14.14.17], encoded by gene A09727, which had the highest expression level at D4, squalene was converted into (S)-squalene-2, 3-epoxide. Lanosterol, a key precursor of triterpenoids, was cyclized from (S)-squalene-2, 3-epoxide by LSS [EC:5.4.99.7], a member of the oxidosqualene-lanosterol cyclase (OSC) family, encoded by gene A01065, which showed the highest expression at D7. Notably, A01065 was the only gene coding for an OSC family member in *P. pulla* according to the transcriptomic annotation. In addition to these key enzymes in the steroid biosynthesis pathway, some other enzymes involved in synthesizing various steroids were also identified in *P. pulla* (Figure 2B). For instance, lanosterol was further modified by lanosterol 14-alpha-demethylase [EC:1.14.14.154] (CYP51, encoded by the genes A05913 and A01905) for the further catalyses to various active substances, and other enzymes, such as methylsterol monooxygenase [EC:1.14.18.9] (MESO1, encoded by the gene A09380), sterol 24-C-methyltransferase [EC:2.1.1.41] (SMT1, encoded by the genes A06292 and A04021), and sterol-4-alpha-carboxylate 3-dehydrogenase [EC:1.1.1.170] (NSDHL, encoded by the gene A07488), catalyzed the early steps in the biosynthesis of calcitetrol, cholesterol, cycloartenol, and fecosterol.

### 2.4. Validation of RNA-Seq Data

Compared with that at D1, the three DEGs, viz. LSS, SQLE and FDFT1, all showed a significantly higher expression level at D4, with LSS also showing a significantly elevated expression level at D7 (Figure S1). The expression patterns of these genes were highly consistent with the RNA-seq (Figure 2A), confirming the validity and reliability of the RNA-seq results.

### 2.5. Metabolome Profiles at Different Growth Times

At four growth time points, a total of 1514 compounds, including steroids, terpenoids, flavonoids and carbohydrates, were identified. Of these, 679 were detected in a positive ion mode and 835 in a negative ion mode, with variable importance in projection (VIP) >1 and *p*-value < 0.05. Among these compounds, 69 steroids and 32 triterpenoids were specifically identified (Table S3). The OPLS-DA analysis revealed that the six biological replicates at D1 were distinctly separated along the central axis from those at D4, D7, and D10 (Figure S2), indicating the high reliability of the metabolomic data generated.

### 2.6. Integrative Analysis of Transcriptome and Metabolome

**Figure 3:**
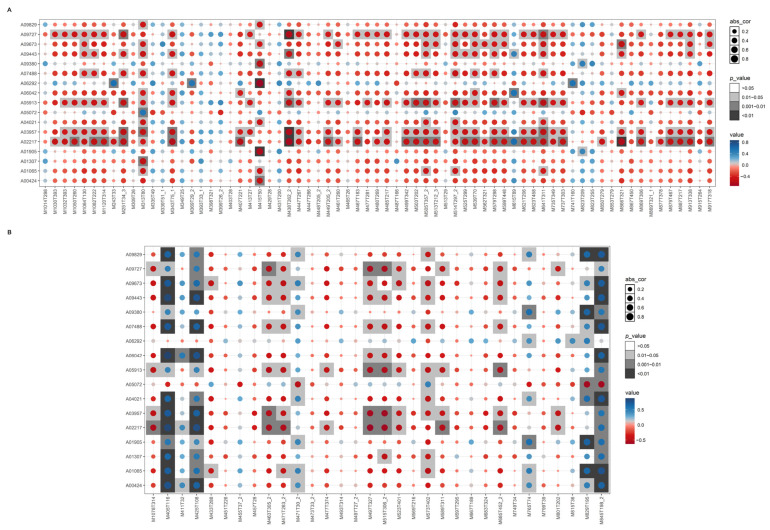
Pearson correlation analysis of DEGs involving the steroids biosynthesis pathways and coding cytochrome P450 monooxygenases with differentially accumulated steroids, steroid derivatives (**A**), and triterpenoids (**B**). The abscissa represents the metabolites, and the ordinate represents the DEGs. The blue and red circles represent positive and negative correlations, respectively, and the color represents the value of correlation coefficient. The abs_cor indicates the absolute value of correlation coefficient via the area of circles.

Cytochrome P450 monooxygenases are known to play a crucial role in steroid biosynthesis [20]. Therefore, in addition to the DEGs involved in the steroid biosynthesis pathway, the DEGs coding for cytochrome P450 monooxygenases (Table S2) were also included in the correlation analyses with steroid metabolites. Generally, these genes exhibited a negative correlation with most steroidal glycosides and alkaloids, with a correlation coefficient (r) > 0.8 and a *p*-value < 0.05 (Figure 3A). This suggests that increased expression of these genes may lead to a reduction in the accumulation of associated metabolites. Notably, several genes, including A09727, A05913, A03957 and A02217, displayed a similar expression pattern in relation to the accumulation of steroids and steroid derivatives.

Furthermore, the correlation of the above-mentioned genes with the accumulation of triterpenoids was also analyzed. Genes such as A09829 and A01065, A09727, A09443, A05913, A03957, A02217, A09380, and A01905 demonstrated similar expression patterns in the accumulation of triterpenoids, with a correlation coefficient (r) > 0.8 and a *p*-value < 0.05 (Figure 3B). Additionally, most genes exhibited a nearly identical expression profile in relation to the accumulation of celastrol (M405T116), lanosterol (M425T108), and Ginsenoside-rg1 (M845T198_2), with positive correlations; and in relation toganoderic acid A (M497T327), evodin (M471T263_2), and pristimerin (M463T305_2), with negative correlations. Specifically, for metabolites, such as celastrol (M405T116), lanosterol (M425T108), and Ginsenoside-rg1 (M845T198_2), the DEGs coding for cytochrome P450 monooxygenases showed expression patterns closely matching those of DEGs involved in the steroid biosynthesis pathway. This indicates that cytochrome P450 monooxygenases play a significant role in the modification of lanosterol and the subsequent biosynthesis of triterpenoids.

### 2.7. Targeted Detection of Celastrol

**Figure 4:**
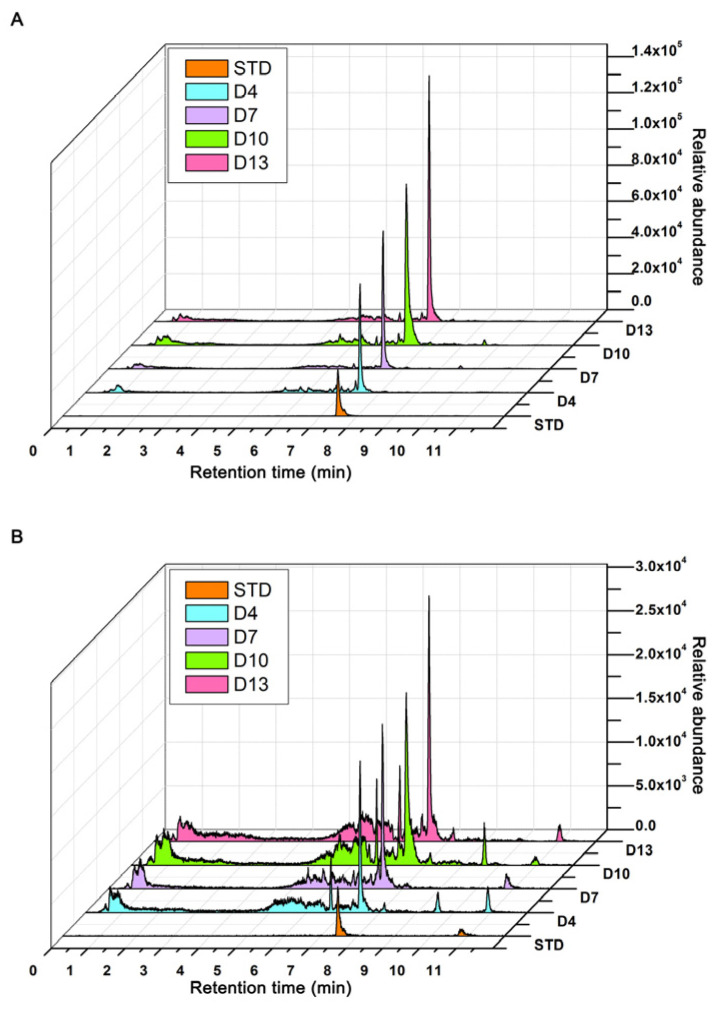
Diagram of LC-MS/MS analysis of celastrol from mycelia of *P. pulla* at four growth stages. The selected ion pairs are 451.4/201.0 (**A**) and 451.4/215.0 (**B**). STD means the standard substance of celastrol.

The relative abundance of celastrol in *P. pulla* at different growth stages during liquid fermentation was determined by comparison with a standard reference substance. No celastrol was detected at D1. However, at subsequent growth stages, the ion pairs 451.4/201.0 (Figure 4A) and 451.4/215.0 (Figure 4B) both indicated that the content of celastrol increased progressively throughout the liquid fermentation process.

### 2.8. WGCNA

**Figure 5:**
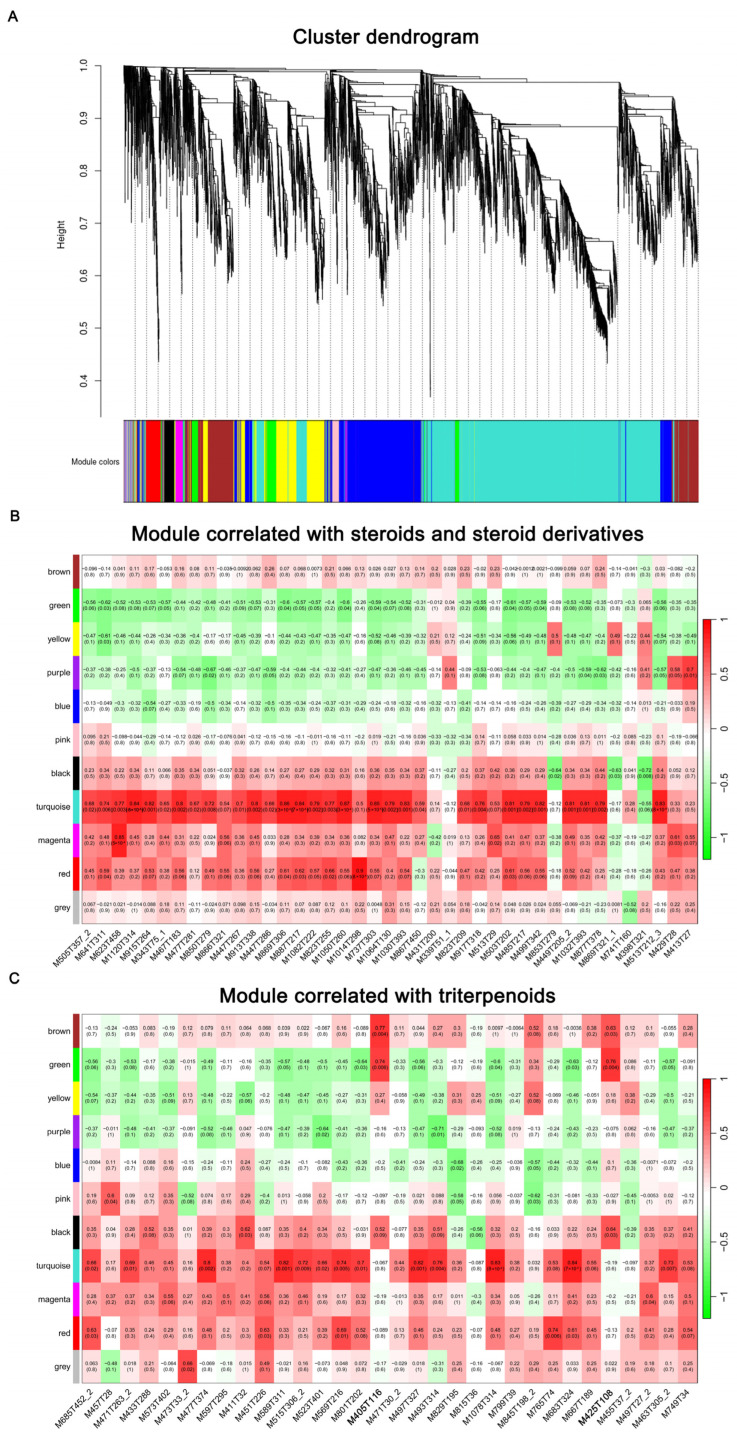
Weighted gene co-expression network analysis. (**A**): Hierarchical cluster dendrogram showing co-expression modules across four growth times. Each leaf of the dendrogram corresponds to one gene, and the leaves constitute 15 modules, labeled with different colors. (**B**): Module correlated with steroids and steroid derivatives. (**C**): Module correlated with triterpenoids. Two triterpenoids, viz. celastrol (M405T116) and lanosterol (M425T108), are indicated in bold font. The numbers in each cell are the correlation coefficient between each gene and metabolite (above) and the corresponding *p*-value (below). Red color indicates a positive correlation, while green color indicates a negative correlation between the gene and metabolite.

To gain further insight into the regulation of the metabolic changes throughout the whole growth process, WGCNA was conducted to investigate the co-expression networks of genes involved in the biosynthesis of steroids and triterpenoids. After filtering out the genes with a low expression level (FPKM < 0.05), the remaining 9677 genes were categorized into 15 distinct modules (Figure 5A). Among these, the turquoise module showed a significant correlation with both steroids and steroid derivatives, as well as triterpenoids, with a correlation coefficient >0.5 and a *p*-value < 0.05 (Figure 5B,C). This suggests that genes within the turquoise module may play a crucial role in the biosynthesis of steroids, steroid derivatives, and triterpenoids. 

The expression patterns of co-expressed genes in the turquoise module were further explored through a soft clustering analysis. Five clusters were revealed, of which four clusters exhibited the highest expression level at D4 and/or D7, while the third cluster displayed an opposite expression pattern (Figure S3A). Additionally, the heatmap also indicated that most of the DEGs in the turquoise module were highly expressed at D4 and D7 (Figure S3B). This expression pattern correlates with the differential accumulation of steroids, steroid derivatives, and triterpenoids, which generally peaked at D4 and D7 (Figure S4). Moreover, the content of triterpenes was found to be highest at D7 and D10, followed by D4, and lowest at D13 (Figure S5). This trend is partially consistent with the expression pattern of the DEGs in the turquoise module.

Although most steroids, steroid derivatives, and triterpenoids were correlated with the turquoise module, two triterpenoids, viz. celastrol and lanosterol, exhibited a closer correlation with the brown and green modules, respectively, with a correlation coefficient > 0.5 and a *p*-value < 0.05 (Figure 5C). Furthermore, unlike the turquoise module, DEGs in the brown (Figure S6) and green (Figure S7) modules generally showed the highest expression levels at D1. 

**Figure 6:**
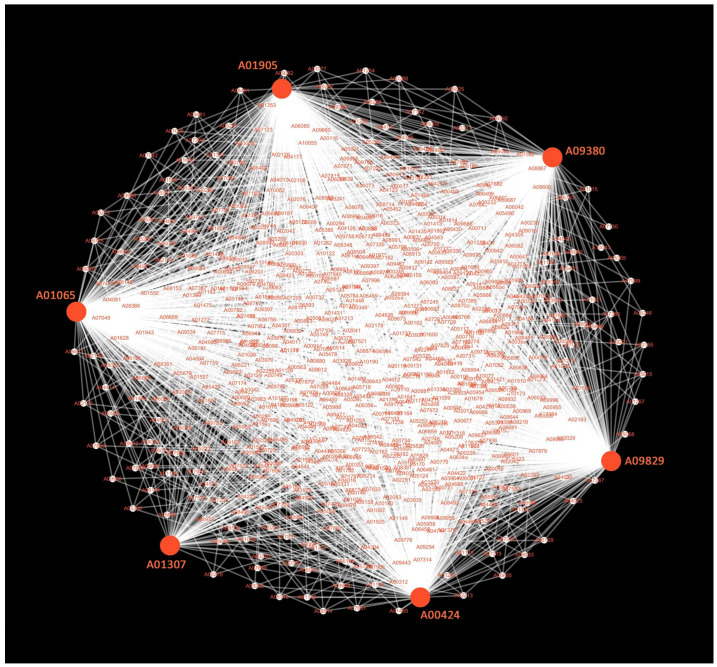
The co-expression network of genes in the brown module mostly correlated with celastrol. The hub genes are highlighted by red color, and the six key hub genes are indicated by larger circles filled with red color (See Table S4 for details).

Given the significant biological activities of celastrol, we examined the genes involved in its biosynthesis by constructing a co-expression network for genes in the brown module (Table S4), which was most strongly correlated with celastrol (Figure 5C). Six key genes were identified with strong correlations, viz. A00424, A01307, A01065, A01905, A09380, and A09829 (Figure 6). Among these, A00424 coded a cytochrome P450 monooxygenase involved in the biosynthesis of steroids, steroid derivatives, and triterpenoids, while the remaining five genes were directly involved in the steroid biosynthesis pathway (Figure 2). In addition, these six key hub genes exhibited high connectivity with genes related to genetic information processing, including ribosomal RNA-processing proteins, ATP-dependent RNA helicases, translation initiation factors, and cell membrane-associated proteins (Figure 6).

## 3. Discussion

*Phylloporia pulla* has a long history of medicinal utilization [19] and has recently been applied for functional food applications [4]. In this study, we further investigate the secondary metabolites of *P. pulla*, with an emphasis on steroids, steroid derivatives, and triterpenoids, as well as the regulatory genes involved in their biosynthesis. The research was conducted through an integrative analysis of transcriptome and metabolome data along with corresponding experimental confirmation.

Steroids are a prominent class of natural organic compounds found in animals, plants, and fungi, with substantial therapeutic value across a range of diseases [21]. Triterpenoids, another diverse group of compounds, are widely distributed among eukaryotes, and many have beneficial properties for human health [22]. In this study, transcriptome data revealed a significant enrichment of the DEGs in the steroid biosynthesis pathway in *P. pulla* based on the transcriptome data. Given that the expression patterns of DEGs are known to regulate the accumulation of specific secondary metabolites under varying growth conditions [23], our findings offer new insights into the biosynthesis of steroids, steroid derivatives, and triterpenoids in *P. pulla*.

To date, few studies have integrated transcriptome and metabolome data for the analysis of steroid metabolic pathways. FDFT1, a key enzyme present in plants, animals, and fungi, plays a crucial role in regulating the biosynthesis of steroids and terpenoids. Overexpression of gene coding FDFT1 can directly enhance the accumulation of these compounds [24,25]. In *P. pulla*, the expression level of the gene A09829, which encodes FDFT1, was higher at the early to middle sampling time. Similarly, other genes involved in the steroid biosynthesis pathway exhibited comparable expression patterns (Figure 2A). Notably, T genes A09829 and A01065 encode two key rate-limiting enzymes, FDFT1 and LSS, respectively [26,27]. Consistent with the high expression levels of these genes, the accumulation of steroids, steroid derivatives, and triterpenoids was notably higher at the mid-point of the sampling period (Figures S4 and S5). This suggests a strong correlation between gene expression and metabolite accumulation of these metabolites. Furthermore, the middle stage appears to be the optimal time for the targeted acquisition of specific secondary metabolites in *P. pulla*, a trend also observed in *S. sanghuang* [14]. While further investigation is required to construct a more comprehensive metabolic pathway, these findings provide a foundation for the targeted extraction of specific secondary metabolites. Additionally, this study highlights an efficient and cost-effective method for producing these medicinal metabolites without the need for long-term liquid fermentation of mycelia, facilitating the accurate and efficient production of steroids, steroid derivatives, and triterpenoids in *P. pulla*.

It is known that some medicinal triterpenoids originally identified in plants have also been associated with fungi. For instance, pristimerin was reported in *Russula flavida* [28], betulinic acid was identified in the *Polyporales* [29], and ginsenoside was involved in biotransformation through liquid fermentation of medicinal fungi [30,31]. In this study, these compounds were also identified for the first time from *P. pulla* through metabolomic analysis. Notably, celastrol, a non-plant-derived compound, was unexpectedly found in *P. pulla* and further confirmed by LC-MS/MS. Celastrol, a friedelane-type triterpene originally isolated from the root of *Tripterygium wilfordii* in 1936 [32], has shown efficacy in treating inflammatory and autoimmune diseases [33], Alzheimer’s disease [34], and cancer [35]. It has significant pharmaceutical potential, but the low production of celastrol limits its large-scale application. By contrast, the mycelia of *P. pulla* grow more rapidly than *T. wilfordii*, potentially offering a more efficient source of celastrol.

In the biosynthesis process of celastrol in *T. wilfordii*, the cyclized triterpene skeleton friedelin is typically catalyzed from the precursor (S)-squalene-2, 3-epoxide by OSCs [36]. Additionally, cytochrome P450 monooxygenases are involved in subsequent oxidation steps [37]. However, the comprehensive biosynthesis pathway of celastrol remains unresolved. In *P. pulla*, the gene A01065, which encodes LSS, a member of the OSC family, was also identified (Table S2). The expression pattern of A01065 showed a strong correlation with celastrol accumulation (Figure 3), suggesting that LSS plays a crucial role in the celastrol biosynthesis in *P. pulla*. Notably, LSS catalyzes the conversion of (S)-squalene-2, 3-epoxide to lanosterol in *P. pulla* (Figure 2), whereas friedelin was produced under the catalysis of LSS in *T. wilfordii*. This divergence is consistent with the known ability of different OSCs to catalyze cyclization reactions leading to various triterpenoid backbones [20]. Furthermore, genes encoding cytochrome P450 monooxygenases involved in the biosynthesis of steroids, steroid derivatives, and triterpenoids (Table S2) also exhibited a high correlation with the celastrol accumulation in *P. pulla*. This supports the hypothesis that *P. pulla* and *T. wilfordii* may share a similar celastrol biosynthesis pathway. 

Although targeted detection of celastrol in *P. pulla* indicates that its content increases with prolonged growth, the current study, which focused on a broad spectrum of medicinal metabolites, did not yield significant amounts of celastrol. The use of appropriate elicitors, such as methyl jasmonate, during *P. pulla* cultivation could potentially enhance celastrol production, analogous to improvements observed in triterpene accumulation in *S. sanghuang* [14] and *Ganoderma applanatum* [38]. Furthermore, elucidating the biosynthetic pathway of celastrol in *P. pulla* will facilitate the regulation of related gene expression, thereby potentially increasing celastrol production. In addition, this study may draw more attention to the utilization and conservation of strategic macrofungal resources [39,40,41].

## 4. Materials and Methods

### 4.1. Strain Cultivation and Genome Sequencing

The fungal stain of *Phylloporia pulla* S-LWZ 20190428-3 used in this study was isolated from the wild fruiting bodies LWZ 20190428-3 growing on living *Pyrus* in Zibo, Shandong Province, China [19]. Both the strain and the specimen are preserved in our lab.

The mycelia of *P. pulla* were harvested after the growth on the sterile cellophane covering potato dextrose agar (PDA) plate culture medium with the addition of 25% sawdust filtrate at 28 °C for 5–7 days. Genomic DNA was extracted from the harvested mycelia using a CTAB rapid plant genome extraction kit-DN14 (Aidlab Biotechnologies Co., Ltd., Beijing, China). PacBio Sequel and Illumina NovaSeq PE150 platforms were used for whole genome sequencing at the Beijing Novogene Bioinformatics Technology Co., Ltd. (Beijing, China). The generated genome sequences were assembled with SMRT Link v5.0.1 [42]. The clean reads were integrated to correct the SNPs and InDels in the draft assembly using Pilon v1.22 [43]. Genome components were predicted using Augustus 2.7 program [44], and then served as a reference genome for transcriptomic analyses. The genome assembly was submitted to the National Microbiology Data Center (https://nmdc.cn/, accessed on 10 October 2024) with the accession number NMDC20292612.

The mycelia of *P. pulla* cultivated in the above-mentioned solid medium were transferred to the liquid fermentation medium (71.951% potato–carrot filtrate, 6.037 g/L glucose, 0.870 g/L peptone, 0.05 g/L KH_2_PO_4_, 0.025 g/L MgSO_4_·7H_2_O and 0.005 g/L ZnSO_4_·7H_2_O) that was optimized using response surface methodology.

### 4.2. RNA Extraction, Sequencing and Data Analyses

Total RNA was extracted from mycelia at D1, D4, D7, and D10, with three biological replicates at each sampling time using TRIzol Reagent (Thermo Scientific, Waltham, MA, USA), following the manufacturer’s protocol, and quantified using a NanoDrop spectrophotometer (Thermo Scientific, Waltham, MA, USA). Then, sequencing libraries were generated using the VAHTS mRNA-seq V3 Library Prep Kit for Illumina (Vazyme Biotech, Nanjing, China). Transcriptome sequencing was performed on a NovaSeq 6000 platform (Illumina, San Diego, CA, USA) by Personal Biotechnology Co., Ltd. (Shanghai, China). The raw reads were preprocessed by removing low-quality reads and adaptor reads using SOAPnuke [45]. Filtered clean reads of transcriptome sequences were mapped to the reference genome of *P. pulla* using HISAT2 (https://daehwankimlab.github.io/hisat2/, accessed on 21 December 2023). 

The gene expression levels were normalized by fragments per kilobase per million fragments (FPKM) [46]. Differentially expressed genes (DEGs) were identified using the DESeq R package, and a *p*-value < 0.05 with a twofold change was considered to be significantly different. A principal component analysis (PCA) was performed to compare the FPKM values of the expressed gene profiles among various sampling times using the Prcomp function in R. To visualize the expression levels of the DEGs in each sample, a volcano map was constructed using the ggplots2 package in R, while Hierarchical clustering and a heatmap were generated using the Pheatmap package in R. DEGs were mapped to the Kyoto Encyclopedia of Genes and Genomes (KEGG) databases to identify their functions and biological processes.

### 4.3. Real-Time Quantitative PCR (qPCR)

To validate the accuracy of transcriptome data, three genes coding key rate-limiting enzymes, viz. lanosterol synthase (LSS), squalene monooxygenase (SQLE) and squalene synthase (FDFT1), were selected for qPCR (Table S1). The RNA extracted for transcriptome analyses from mycelia at D1, D4, D7, and D10 were used. Reactions were performed on Bio-Rad CFX96 real-time PCR detection system with the SYBR qPCR Master Mix (Accurate Biology, Changsha, China). For each gene, a fold change value was estimated in terms of threshold cycles according to the 2^−ΔΔCT^ method with three biological replicates and three technical replicates for each sample. The beta-tubulin gene was chosen as the endogenous reference gene.

### 4.4. Metabolome Identification and Quantification

The mycelia at D1, D4, D7, and D10, with six biological replicates for each sampling time, were harvested for non-targeted metabolome identification. A mixture of methanol, acetonitrile, and water at the ratio of 2:2:1 (*v*/*v*/*v*) was added into each sample for extraction for 60 min. Then, samples were centrifuged at 14,000 rpm at 4 °C for 10 min, and the supernatants were collected and dried in a vacuum. The dried samples were resuspended with 100 μL of acetonitrile and water at the ratio of 1:1 (*v*/*v*), and then centrifuged at 14,000 rpm at 4 °C for 15 min after shaking using a vortex mixer. 

Thereafter, the metabolites were determined by a 1290 Infinity Ultra high-pressure liquid chromatograph (Agilent) linked to a Triple TOF 6600 Mass spectrometer (Applied Biosystems/SCIEX) with a chromatographic column (Waters, ACQUITY UPLC^®^ BEH Amide column, 1.7 µm, 2.1 mm × 100 mm). The analysis conditions were as follows: column temperature of 25 °C, injection volume of 2 μL, and flow rate of 0.5 mL/min. The mobile phases were water (H_2_O, phase A) and acetonitrile (0.1% formic acid, phase B). The gradient elution program was as follows: 95% B (0–0.5 min), B linearly decreasing from 95% to 65% (0.5–7 min), B linearly decreasing from 65% to 40% (7–8 min), B maintaining at 40% (8–9 min), B increasing from 40% to 95% (9–9.1 min), and B maintaining at 95% (9.1–12 min).

The qualitative method of metabolites was to match the exact molecular weight with data in the online databases of METLIN, in which the adduct manner was selected as [M+H]^+^ and [M+Na]^+^ in positive mode and [M-H]^−^ and [M+FA-H]^−^ in negative mode with the mass error value being 20 PPM. To screen the differentially accumulated metabolites between D1 and another three sampling times, each pair was analyzed by the Student’s *t*-test and orthogonal projections to latent structures discriminant analysis (OPLS-DA). Afterwards, the metabolites with a variable importance in projection values >1 and *p*-value < 0.05 were selected as the differentially accumulated metabolites. Finally, the differentially accumulated metabolites from positive and negative modes were combined to conduct KEGG pathway analysis.

The correlation between the expression patterns of genes and the differential accumulations of metabolites was estimated using the Pearson algorithm in psych R package v4.2.1. 

### 4.5. Determination of Triterpenes

The triterpene fraction was extracted from dried mycelia harvested at D1, D4, D7, D10, and D13 using an ethanol extraction method. In brief, 2 g of mycelium powder were mixed with anhydrous ethanol at a 1:25 (*w*/*v*) ratio, stirred, and then subjected to ultrasonic agitation (140 W, 42 kHz) for 45 min. Subsequently, the mixture was centrifuged at 4500 rpm for 10 min. The supernatant was transferred to a 100 mL volumetric flask and brought up to volume with anhydrous ethanol. The content of triterpene was determined using the vanillin–perchloric acid assay, as described in the Pharmacopoeia of the People’s Republic of China (Chinese Pharmacopoeia Commission, 2020), with oleanolic acid as the standard.

### 4.6. Targeted Detection of Celastrol by LC-MS/MS

The dried mycelia harvested at D1, D4, D7, D10, and D13 were separately ground into powder for ultrasonic extraction with ethyl acetate (40 °C, 100 Hz, 2 h) for two times. The liquid extracts were concentrated by rotary evaporation. After being redissolved with methanol, the samples were eluted with a Millipore filter (polytetrafluoroethylene, 0.22 µm; Anpel) and the resulting solution was subjected to LC-MS/MS analysis. A 6500 QTRAP triple quadrupole mass spectrometer (Applied Biosystems/SCIEX) coupling ExionLCTM system (Applied Biosystems/SCIEX) was used to detect celastrol with 0.1% formic acid and 100% acetonitrile as mobile phases A and B, respectively. Chromatographic separations were accomplished on a Phenomenex Gemini^®^ NX-C18 100 Å LC column (3 µm, 50 mm × 2 mm) maintained at 40 °C with 10 µL of sample volume at a flow rate of 0.3 mL/min. The liquid gradient was as follows: 20% B (0–3 min), 20–90% B (3–6 min), 90% B (6–9 min), 90–20% B (9–10 min), and 20% B (10–12 min). MS/MS detection was carried out by electrospray ionization and multiple reaction monitoring (MRM) scans under positive or negative ion modes, respectively. In MRM transitions, one precursor (*m*/*z*: 451.400) and two product ions (*m*/*z*: 201.000 and 215.000) were selected for quantitative and qualitative analyses [47]. Mapping to the standard substance of celastrol (Jinming Biotechnology Co., Ltd., Beijing, China), each analyte was identified by comparing with transitions (451.400 → 201.000; 451.400 → 215.000) and retention time (7.48 min). The concentration was determined by the peak area in the MRM chromatogram with MultiQuant software (Version 3.0.2; Applied Biosystems/SCIEX).

### 4.7. Weighted Gene Co-Expression Network Analysis

Weighted gene co-expression network analysis (WGCNA) package [48] was used to reveal the genes related to the biosynthesis of steroids and triterpenoids. All expressed genes and differentially accumulated metabolites detected at D1, D4, D7 and D10 were included in the network analysis. The co-expression modules were obtained using automatic network construction function (blockwiseModules) with default parameters, except that the soft threshold power was 18, TOMtype was unsigned, mergeCutHeight was 0.25 and minModuleSize was 30. The initial clusters were merged with eigengenes. The eigengene value of each module was used to search the correlation with differentially accumulated metabolites. The fuzzy clustering method in the Mfuzz R package [49], which is insensitive to noise, was performed to explore the expression pattern of the DEGs in co-expression modules. The expression pattern was also shown in the form of a heatmap that was generated using the Pheatmap package in R. The co-expression network of modules was visualized using Cytoscape v3.9.1.

### 4.8. Data and Statistical Analysis

All above-mentioned experiments were performed in triplicate. The data obtained from screening fermentation strains are presented as a mean ± standard deviation. Statistical significance between different groups was determined using one-way of variance (ANOVA). The statistical analyses were conducted using SPSS (v17.0) software, where *p*-value < 0.05 was considered to be statistically significant.

## 5. Conclusions

In summary, this study provides, for the first time, multiomics insights into the applied potential of bioactive secondary metabolites in *P. pulla*. Genes that are strongly correlated with steroids, steroid derivatives, and triterpenoids exhibited elevated expression levels during the early to middle stages of growth. Correspondingly, these metabolites were predominantly accumulated at the middle stage of growth. This data is essential for the targeted utilization of the bioactive metabolites produced by *P. pulla*. 

## Data Availability

The data that support the findings of this study are available from the corresponding author upon reasonable request.

## Supplementary Materials

The following supporting information can be downloaded at: https://www.mdpi.com/article/10.3390/ijms252011070/s1.
