# Supplementary figures and images for: Transcriptome and Metabolome Reveal Accumulation of Key Metabolites with Medicinal Properties of Phylloporia pulla
Source: Int J Mol Sci. 2024 Oct 15;25(20):11070. doi: 10.3390/ijms252011070 (PMC11507218; doi:10.3390/ijms252011070)

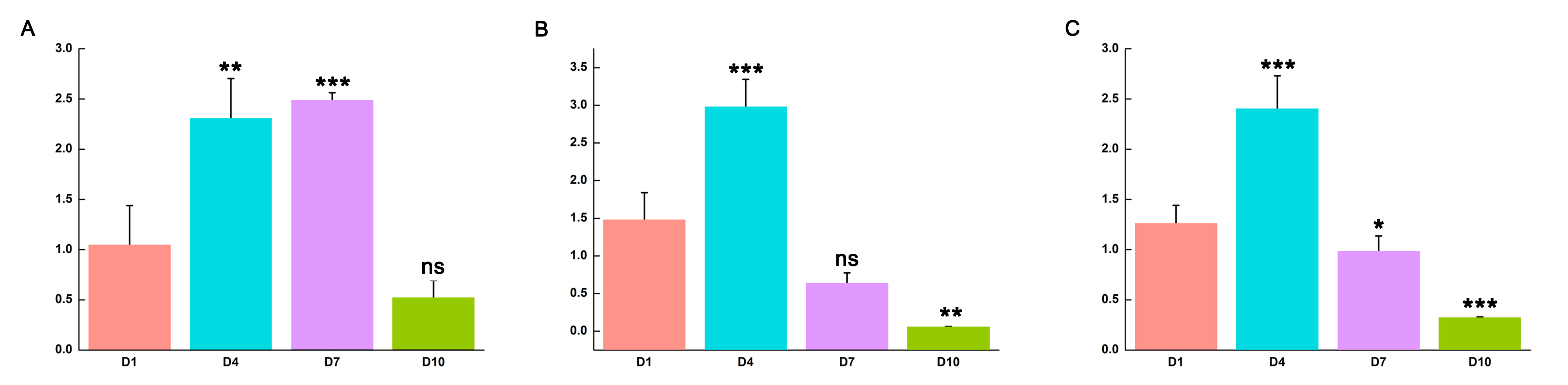

Supplement: Supplementary file 1 [file ijms-25-11070-s001.zip › Figure S1.jpg]

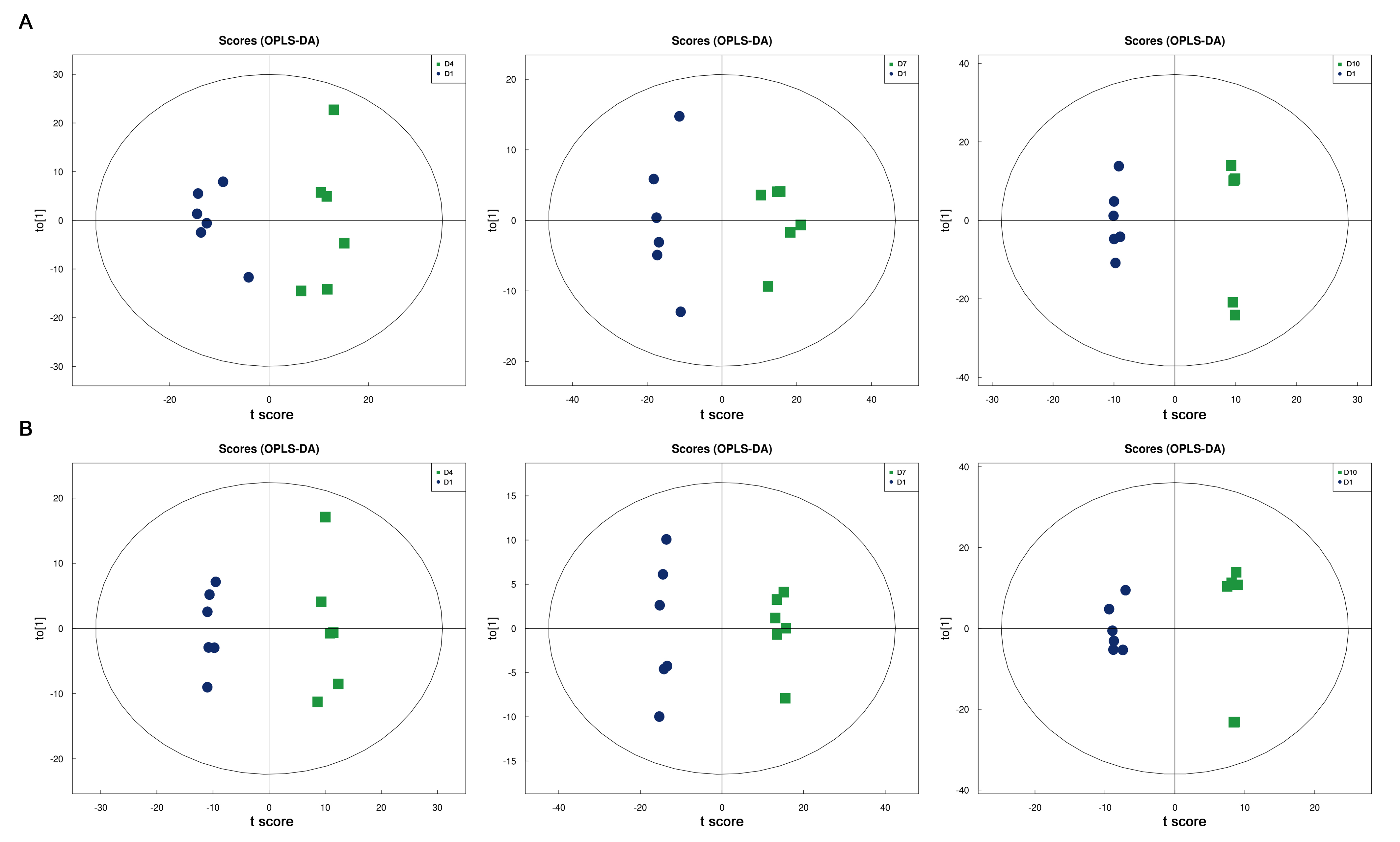

Supplement: Supplementary file 1 [file ijms-25-11070-s001.zip › Figure S2.jpg]

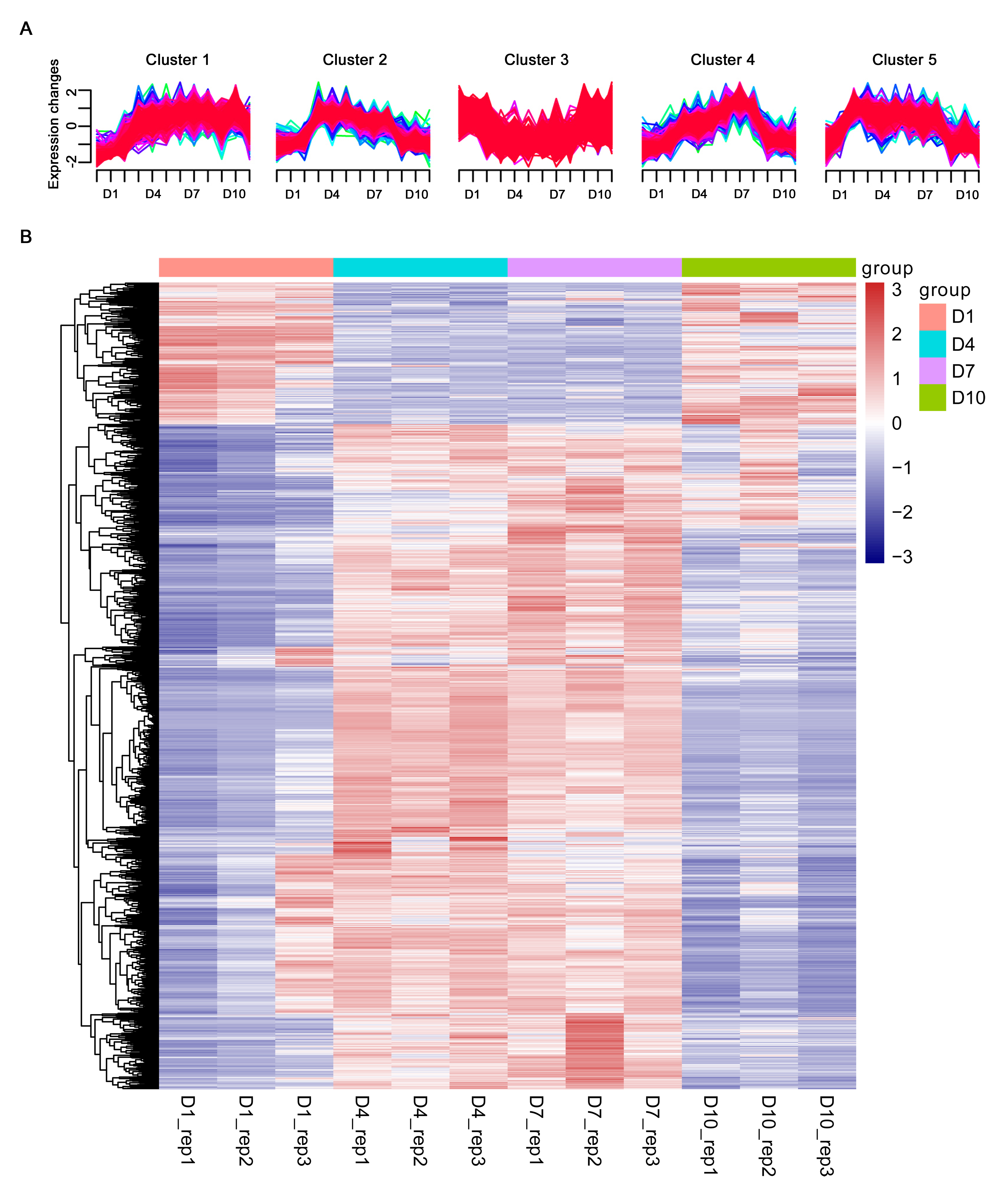

Supplement: Supplementary file 1 [file ijms-25-11070-s001.zip › Figure S3.jpg]

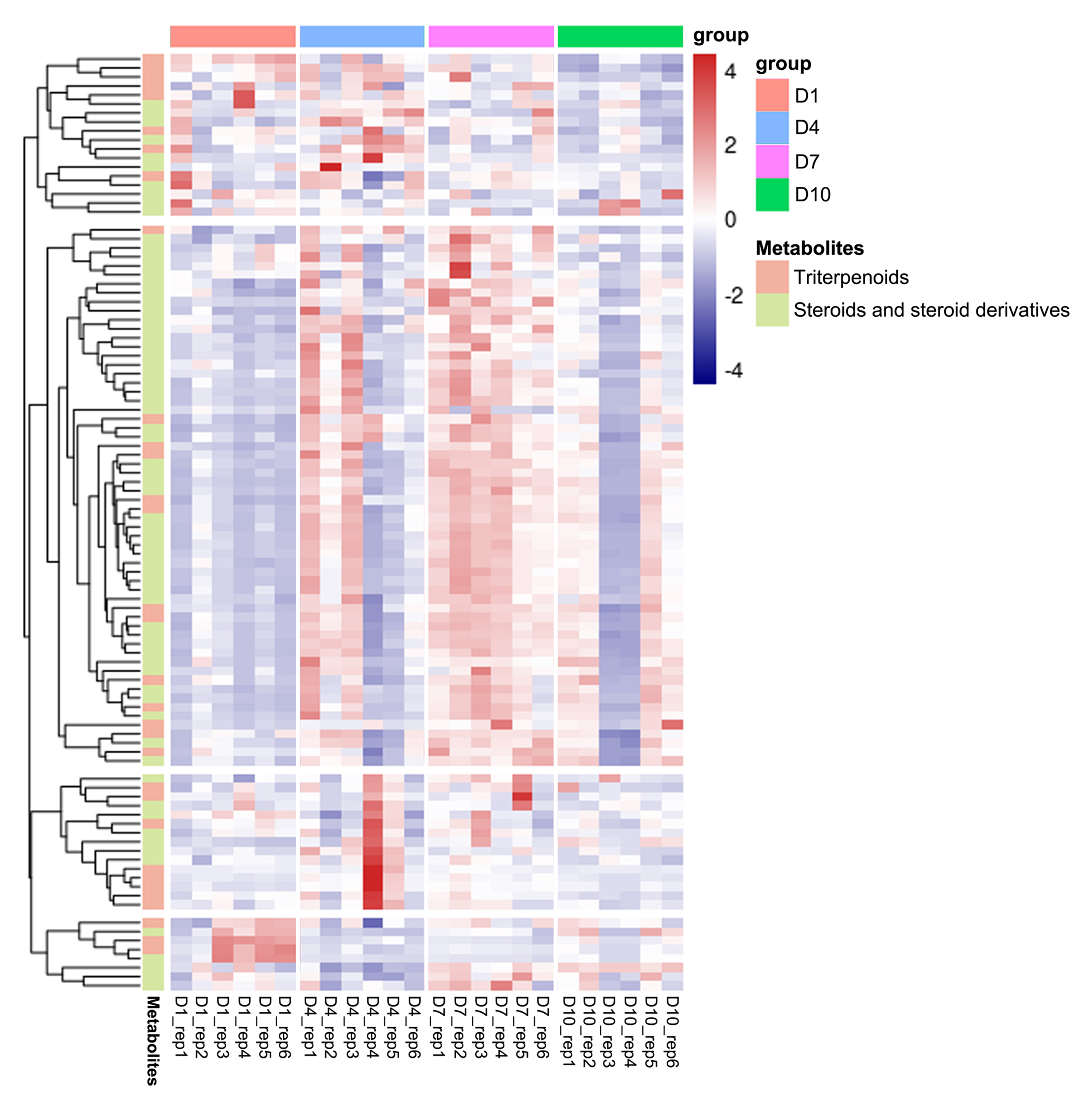

Supplement: Supplementary file 1 [file ijms-25-11070-s001.zip › Figure S4.jpg]

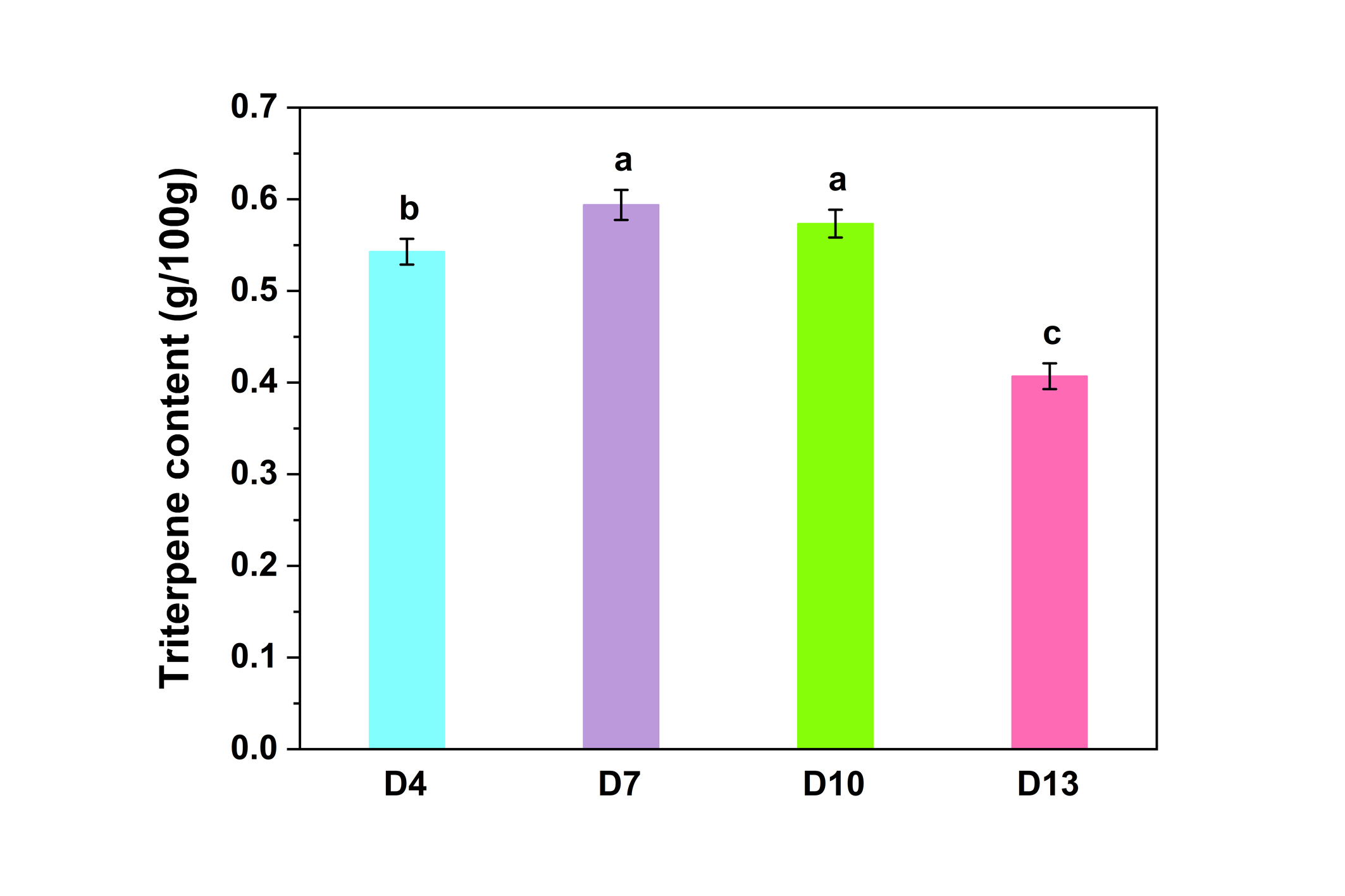

Supplement: Supplementary file 1 [file ijms-25-11070-s001.zip › Figure S5.jpg]

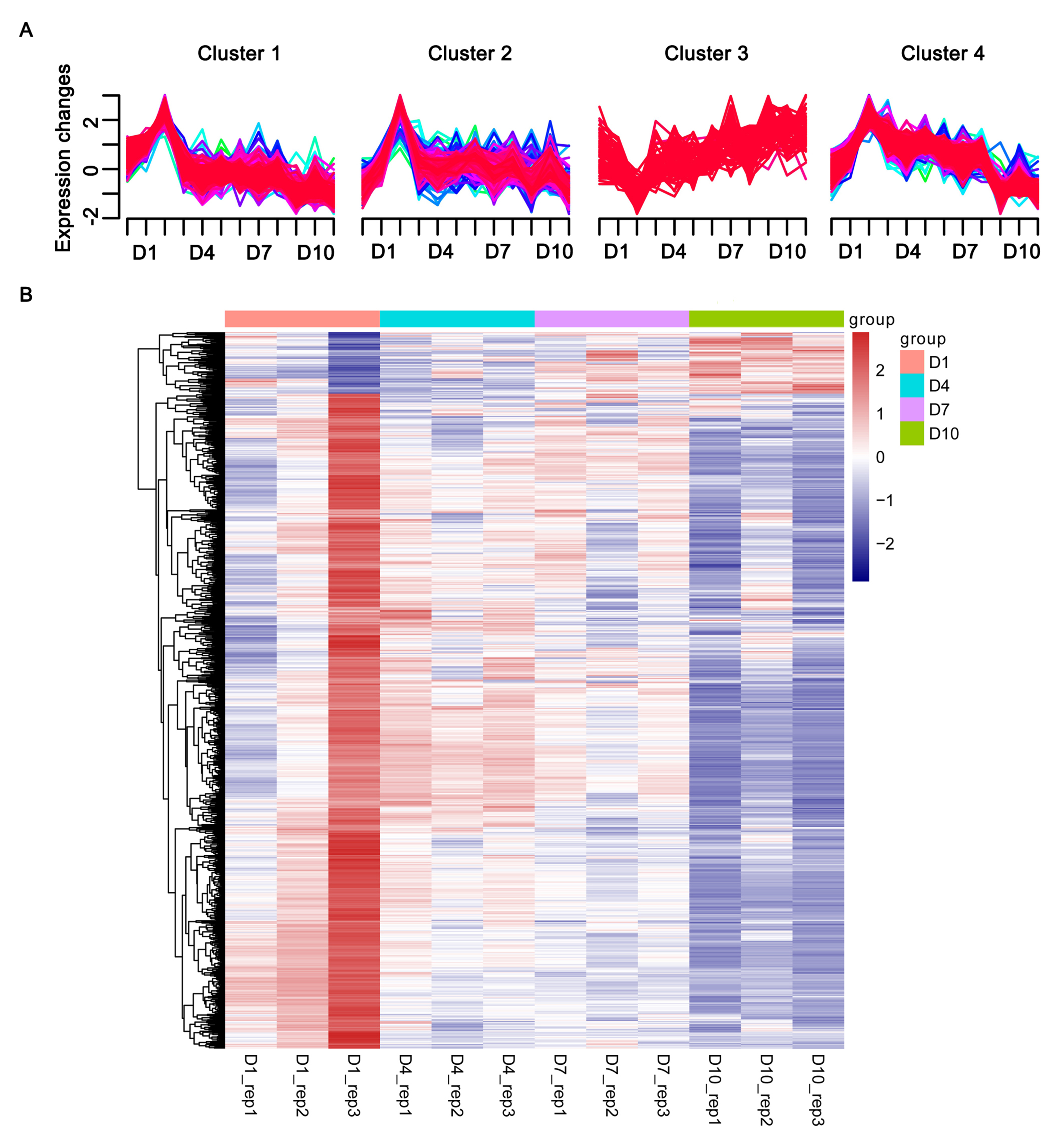

Supplement: Supplementary file 1 [file ijms-25-11070-s001.zip › Figure S6.jpg]

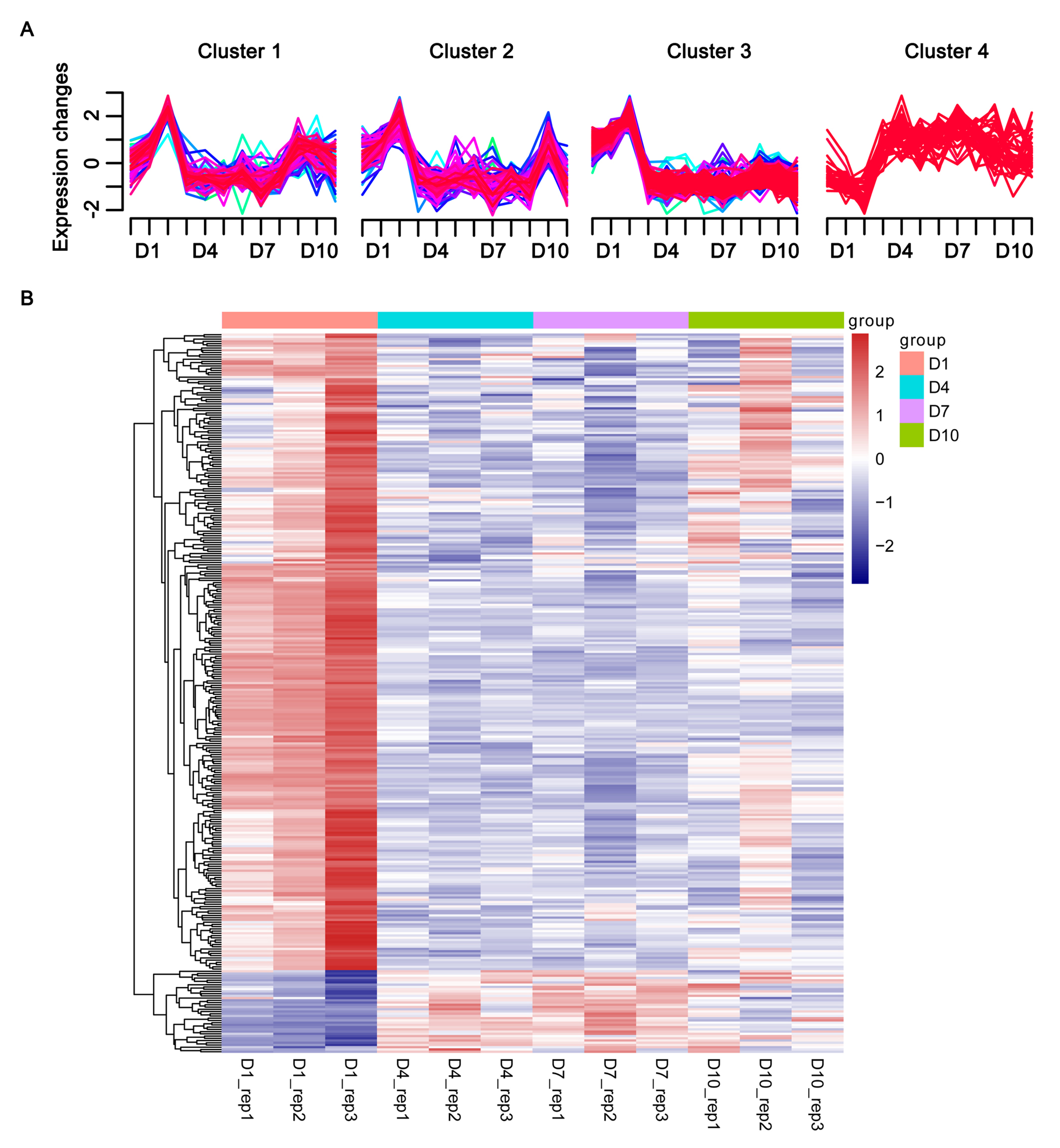

Supplement: Supplementary file 1 [file ijms-25-11070-s001.zip › Figure S7.jpg]
